# Nutritional Modulation of the Endogenous Antioxidant System in the Brain–Gut Axis Following Traumatic Brain Injury

**DOI:** 10.3390/nu17213404

**Published:** 2025-10-29

**Authors:** Nicla Tranchida, Francesca Inferrera, Rosalba Siracusa, Daniela Impellizzeri, Ramona D’Amico, Rosanna Di Paola, Marika Cordaro, Roberta Fusco

**Affiliations:** 1 Department of Chemical, Biological, Pharmaceutical and Environmental Sciences, University of Messina, Viale F. Stagno D’Alcontres 31, 98166 Messina, Italy; 2 Department of Medicine and Surgery, “Kore” University of Enna, 94100 Enna, Italy; 3 Department of Veterinary Sciences, University of Messina, Viale SS Annunziata, 98168 Messina, Italy; 4 Department of Biomedical, Dental and Morphological and Functional Imaging, University of Messina, Via Consolare Valeria, 98125 Messina, Italy

**Keywords:** neuroinflammation, gut health, functional food, nutrition

## Abstract

**Background/Objectives:** Traumatic brain injury (TBI) disrupts both the intestinal epithelium and blood–brain barrier (BBB), contributing to oxidative stress, neuroinflammation, and behavioral impairments. *Vitis vinifera* leaf (VVL) extract possesses antioxidant and anti-inflammatory properties, but its protective effects on the brain–gut axis following TBI remain unclear. This study aimed to evaluate whether VVL supplementation preserves barrier integrity and improves neurobehavioral outcomes after TBI. **Methods:** A murine model of TBI was used, with animals receiving daily oral supplementation of the VVL extract. Neurobehavioral performance was assessed through behavioral testing, while histopathological examinations, biochemical assays, and gene expression profiling were performed to evaluate neuronal and intestinal integrity, antioxidant defense, and inflammatory responses. **Results:** VVL supplementation significantly alleviated anxiety- and depression-like behaviors and preserved the structural integrity of neuronal and intestinal tissues. Antioxidant defense mechanisms were strengthened, as shown by increased catalase and superoxide dismutase activities, together with upregulation of Nrf2 and HO-1 expression. Tight junction proteins, including ZO-1 and occludin, were upregulated in both brain and gut tissues, reflecting improved barrier integrity. Furthermore, VVL markedly reduced pro-inflammatory cytokine expression. **Conclusions:** VVL extract confers dual protection of the gut and brain barriers after TBI by enhancing endogenous antioxidant defenses, maintaining tight junction integrity, and suppressing inflammation. These findings suggest that VVL may represent a natural therapeutic strategy to mitigate oxidative stress, neuroinflammation, and behavioral dysfunctions associated with TBI.

## 1. Introduction

Traumatic brain injury (TBI) remains a major global health burden, with an estimated 69 million new cases annually, predominantly caused by falls, road traffic accidents, and sports injuries [1,2]. The pathophysiological evolution of TBI is typically categorized into two phases: the primary injury, representing the immediate mechanical insult to brain parenchyma (e.g., cortical contusion, axonal shearing, intracerebral hemorrhage), and the secondary injury, which unfolds over hours to weeks after the initial trauma [3]. This secondary phase comprises a complex cascade of molecular and cellular events, including excitotoxicity, mitochondrial dysfunction, calcium overload, oxidative and nitrosative stress, neuroinflammation, apoptosis, and blood–brain barrier (BBB) breakdown [4]. These processes drive neuronal loss, glial activation, and disruption of neurovascular coupling, ultimately impairing central nervous system (CNS) homeostasis.

Clinical assessment of TBI severity often employs the Glasgow Coma Scale (GCS), with scores of 13–15, 9–12, and <9 indicating mild, moderate, and severe injuries, respectively [5,6,7]. Although most hospital-presenting cases are mild TBIs (>90%), they can still lead to persistent cognitive and neuropsychiatric sequelae [7]. The Full Outline of Unresponsiveness (FOUR) score provides an alternative evaluation, particularly in intubated patients [8].

Among molecular mediators of secondary injury, oxidative stress is a key driver of mitochondrial dysfunction, BBB disruption, and neuronal degeneration [9,10]. The nuclear factor erythroid 2–related factor 2 (Nrf2) pathway is a master regulator of antioxidant defense, controlling the expression of cytoprotective genes such as heme oxygenase-1 (HO-1) [11,12,13]. Upon activation, Nrf2 translocate to the nucleus, binds antioxidant response elements (AREs), and induces transcription of redox-regulating enzymes, restoring cellular equilibrium and mitigating inflammatory signaling [14,15,16,17].

Natural phytochemicals with Nrf2-activating properties have gained attention for their multi-target neuroprotective effects and favorable safety profiles [18,19,20]. *Vitis vinifera* leaves (VVLs), a byproduct of one of the world’s most widely cultivated fruit crops, are rich in polyphenols—including catechins, flavanols, procyanidins, anthocyanins, gallic acid, and resveratrol—that exhibit potent antioxidant, anti-inflammatory, and barrier-protective effects [21,22,23,24]. These compounds can enhance endogenous antioxidant capacity, suppress microglial activation, and modulate cytokine-mediated neurotoxicity through the Nrf2/HO-1 axis [25].

Recent research has also highlighted the gut–brain axis as a critical regulator of neurological outcomes after injury [26,27,28]. Preservation of intestinal barrier integrity can reduce systemic inflammation, thereby preventing peripheral immune activation from exacerbating CNS pathology. Polyphenols, particularly resveratrol, have been shown to upregulate tight junction proteins such as Zonula Occludens-1 (ZO-1) and occludin, strengthening gut barrier function and indirectly supporting BBB stability [29,30]. Tight junctions are essential for maintaining intestinal barrier integrity. When ZO-1 and occludin are upregulated, the tight junctions are reinforced, resulting in improved gut barrier function and a reduction in intestinal permeability, which is often referred to as “leaky gut”. This strengthening of the barrier prevents the translocation of bacteria and luminal toxins into circulation, thereby offering protection against inflammation and gut dysbiosis [31].

Given the interconnected roles of oxidative stress, barrier dysfunction, and systemic inflammation in TBI progression, we hypothesize that VVL may act as a dual-barrier modulator, simultaneously protecting gut and brain integrity while activating antioxidant defense pathways. This study investigates the chronic effects of VVL supplementation in a murine TBI model, focusing on behavioral outcomes, oxidative stress modulation, and preservation of gut–brain homeostasis as an integrated therapeutic strategy.

## 2. Materials and Methods

### 2.1. Animals

Eight-week-old male CD1 mice (18–24 g; Envigo, Milan, Italy) were used in this study. All procedures were conducted in accordance with the European Directive 2010/63, the Italian legislative decree D.Lgs 26/2014, and the ARRIVE guidelines. Experimental protocols received prior approval from the University of Messina Animal Care and Use Committee (OPBA).

### 2.2. Trauma Induction

Traumatic brain injury in mice was produced through a controlled cortical impact (CCI) approach. Briefly, a craniotomy was made over the right cortex, located between the bregma and lambda and extending laterally from the midline toward the coronal suture. Once the bone flap was gently lifted and widened, the intact dura mater was impacted using a stereotaxic CCI device (Impact One™, Leica Microsystems, Milan, Italy). The instrument was adjusted to induce a moderate brain lesion with an impactor diameter of 4 mm, a penetration depth of 3 mm, and a velocity of 1.5 m/s. After impact, the incision on the scalp was sutured, and a 2% lidocaine gel was topically applied to provide postoperative pain relief [32].

### 2.3. Experimental Design and Groups

Three experimental groups, each consisting of five animals, were assigned at random with the technique of “simple randomization” [33]:

- Sham + vehicle: Mice underwent the surgical procedures without cortical impact and received oral vehicle administration.
- Sham + VVL: Mice underwent the surgical procedures without cortical impact and received oral *Vitis vinifera* leaf (VVL) extract.
- TBI + vehicle: Mice were subjected to controlled cortical impact (CCI) and treated with oral vehicle (saline).
- TBI + VVL: Mice received CCI as in the TBI + vehicle group, followed by daily oral supplementation of VVL extract (250 mg/kg in saline), which was initiated 1 h post-injury.

The VVL dose was selected based on previously published studies [34,35,36,37]. Behavioral assessments were conducted on days 1, 7, 14, and 21 post-injury. On day 28, animals were sacrificed, and brain and intestinal tissues were collected for histological and biochemical analyses.

As no significant differences were observed between the Sham + vehicle and Sham + VVL groups, only the Sham + VVL group is presented throughout the manuscript and referred to as “Sham”.

### 2.4. Histological Examination

At the end of the experimental procedures, brain and intestinal tissues were harvested and immersed in 10% of neutral-buffered formalin (prepared in phosphate-buffered saline, PBS) at room temperature for fixation. The samples were then processed through a graded series of ethanol for dehydration, embedded in paraffin, and sliced into 5 μm sections. For brain histological evaluation, the paraffin sections were deparaffinized with xylene, stained with hematoxylin and eosin (H&E), and visualized using a Leica DM6 light microscope equipped with the LAS X Navigator software 3.7.6 (Leica Microsystems SpA, Milan, Italy) [38]. Neuronal damage was quantified by counting eosinophilic. Cells were identified based on shrunken morphology, cytoplasmic eosinophilia, and nuclear pyknosis., and gray matter pathology was scored on a 6-point scale: 0 = no lesion; 1 = 1–5 eosinophilic neurons; 2 = 5–10 eosinophilic neurons; 3 ≥ 10 eosinophilic neurons; 4 = small infarction (<1/3 gray matter area); 5 = moderate infarction (1/3–1/2 gray matter area); and 6 = large infarction (>1/2 gray matter area). For each animal, three non-overlapping fields per region were analyzed at 400× magnification across three consecutive coronal sections. Scores from all brain sections per animal were averaged to obtain a final histopathological score [38]. Analysis was performed by an investigator blinded to treatment groups.

For intestinal evaluation, fixed tissues were processed and stained with H&E, then examined under the same imaging system. Histopathological assessment was performed in five predefined categories: inflammation, epithelial damage, edema, goblet cell loss, and mucosal hyperplasia. Each parameter was graded on a 0–4 scale according to severity (0 = absent; 1 = mild; 2 = moderate; 3 = marked; 4 = severe), with the sum of all categories yielding a maximum total score of 20. Additionally, morphometric analysis was conducted as previously described [39], and measurements from digital brightfield images were made using dedicated software [40]. Measurements were obtained on digital images using the ImageJ 1.54g (NIH, Bethesda, MD, USA) after calibration with a stage micrometer; at least three non-overlapping fields per region and three regions per animal were analyzed. All scoring and measurements were performed by a blinded investigator, and data were expressed as mean ± SEM for statistical analysis.

### 2.5. Morris Water Maze (MWM)

The Morris water maze test was conducted in a circular pool measuring 152 cm in diameter and 60 cm in height, filled with water maintained at 23 °C to a depth of 30 cm. A hidden escape platform (10 cm in diameter) was positioned in one quadrant of the pool, with its surface submerged 2 cm below the water level and kept in the same location throughout the training phase. To minimize external visual cues, the tank was surrounded by a white curtain, and four distinct black geometric shapes were attached to its inner surface as spatial references. Each mouse underwent one training session per day for four consecutive days. Twenty-four hours after the final training session, a probe trial was conducted. During this test, the proportion of distance traveled and the time spent within the target quadrant were recorded and analyzed [41]. In the Morris water maze, progressive reduction in escape latency across training days reflects spatial learning ability, while the probe trial, in which the platform is removed, assesses memory retention by measuring preference for the target quadrant and former platform location.

### 2.6. Elevated Plus Maze Test (EPM)

The elevated plus maze (EPM) consisted of two open arms (25 × 5 × 0.5 cm) and two closed arms (25 × 5 × 16 cm) arranged around a central platform (5 × 5 × 0.5 cm). Memory acquisition was evaluated on day 27 following TBI (test session). Each mouse was placed individually at the distal end of an open arm, oriented toward the open edge of the maze. The time taken by the animal to move from the open arm to one of the enclosed arms was recorded as the initial acquisition latency (IAL). After measuring the IAL, the animal was allowed to explore the maze freely for 20 s before being returned to its home cage. If the mouse failed to enter a closed arm within 90 s, it was gently guided into one, and the latency was recorded as 90 s. Memory retention was assessed 24 h later (day 28 after TBI; re-test session) by recording the retention transfer latency (RTL) under the same conditions. Variations between the test and re-test latencies were interpreted as indicators of experience-dependent modulation of memory processes [42,43,44].

### 2.7. Open Field Test (OF)

Locomotor activity and anxiety-like behavior were assessed using the open field test. The apparatus consisted of a transparent Plexiglas box (50 × 50 cm) with the floor divided into 16 equal squares, including four central and twelve peripheral zones. Each mouse was placed gently in the center of the arena, and its behavior was recorded for 5 min. Movement was quantified by counting the number of line crossings, defined as the animal moving all four paws from one square into an adjacent one. Both the total number of crossings and the duration spent in the central area were measured and analyzed as indicators of exploratory activity and anxiety-related behavior [45].

### 2.8. Evaluation of Neurological Impairment

Neurological function was evaluated using the neurological severity score (NSS) described by Chen et al., which is a 10-point composite scale assessing motor activity, reflexes, balance, and exploratory behavior. The battery consists of ten simple tasks, including exiting from a circle, forelimb extension, hindlimb placing, beam balance, beam walking on different widths, startle reflex, spontaneous activity, walking ability, and general coordination. Each failed task is scored as one point, giving a total score range of 0–10, where 0 indicates normal performance and 10 reflects severe neurological impairment. Assessments were performed by an investigator blinded to the experimental groups to minimize bias [46].

### 2.9. Measurement of Edema (Brain Water Content)

At 28 days post-TBI, the animals were sacrificed to assess brain water content as an indicator of cerebral edema. The cerebral cortices, excluding the cerebellum, were carefully dissected, and the ipsilateral and contralateral hemispheres were weighed separately to obtain their wet weights. The samples were then dried at 60 °C for 72 h, after which the dry weights were recorded to calculate the water content [47]. Water content was calculated in the ipsilateral hemisphere as follows:

Water content (%) = (Wet weight − dry weight)/wet weight × 100

### 2.10. Real-Time PCR

Total RNA was isolated using the RNeasy Mini Kit (Qiagen, Milan, Italy) following the manufacturer’s instructions, and its concentration and purity were determined using a NanoDrop Lite spectrophotometer (Thermo Fisher Scientific, Waltham, MA, USA). Complementary DNA (cDNA) was synthesized from the extracted RNA with the iScript Reverse Transcription Supermix (Bio-Rad, Hercules, CA, USA) according to the provided protocol. For quantitative real-time PCR, 1 μL of cDNA was amplified using the SYBR Green detection method on an Applied Biosystems Real-Time PCR System (Thermo Fisher Scientific, Waltham, MA, USA). The analyzed target genes included Zonula Occludens-1 (ZO-1), Occludin, Nuclear factor erythroid 2–related factor 2 (Nrf2), and Heme Oxygenase-1 (HO-1). Glyceraldehyde 3-phosphate dehydrogenase (GAPDH) served as the internal reference for normalization of gene expression levels. Each reaction was performed in triplicate, and negative controls containing RNA instead of cDNA were included to confirm the absence of genomic DNA contamination. The following primer was used for rt-PCR:

Zo-1
Forward 5′-GATAGTTTGGCAGCAAGAGATGGTA-3′
Reverse 5′-AGGTCAGGGACGTTCAGTAAGGTAG-3′
Nrf2
Forward: 5′-AAAATCATTAACCTCCCTGTTGAT-3′
Reverse: 5′-CGGCGACTTTATTCTTACCTCTC-3′
HO-1
Forward: 5′-TAAGACCGCCTTCCTGCTCA-3′
Reverse: 5′-CTGACGAAGTGACGCCATCT-3′
occludin
Forward 5′-CCTTCTGCTTCATCGCTTCCTTA-3′
Reverse 5′-CGTCGGGTTCACTCCCATTAT-3′
GAPDH:
Forward 5′-TGAACGGGAAGCTCACTGG-3′
Reverse 5′-GCTTCACCACCTTCTTGATGTC-3′.

### 2.11. Biochemical Analysis

SOD activity was measured with the Abcam kit (ab285309) according to the manufacturer’s protocol (Abcam, Cambridge, UK). In brief, tissue samples (brain and gut) were homogenized on ice in cold PBS and centrifuged at 12,000× *g* for 10 min at 4 °C; supernatants were collected and total protein concentration determined by BCA. The kit assay is based on the generation of superoxide (via xanthine/xanthine oxidase) that reduces a tetrazolium salt; SOD activity inhibits formation of the formazan dye in a dose-dependent manner. Absorbance was read using a microplate reader at the wavelength specified by the kit, and SOD activity was calculated against the supplied standards and expressed as U per mg protein. All samples were assayed in technical duplicate and blank-corrected.

Reduced glutathione (GSH) was quantified using the Abcam fluorescent assay kit (ab239727) following the manufacturer’s instructions. Briefly, tissue homogenates were prepared as above and, where required by the kit, deproteinized (per kit protocol) prior to assay. GSH reacts with a thiol-reactive probe to form a stable fluorescent product; fluorescence was measured at the excitation/emission wavelengths recommended by the kit (Ex/Em as provided by Abcam), and concentrations were interpolated from a standard curve. Final GSH values were normalized to protein (µmol or nmol GSH per mg protein). Assays were run in duplicate.

Catalase activity was measured using the Cusabio kit (CSB-E14190m) following the supplier’s instructions (CUSABIO Technology LLC, Houston, TX, USA). The assay quantifies catalase-mediated decomposition of H_2_O_2_: after incubation with sample, residual H_2_O_2_ is reacted with a chromogenic/peroxidase reagent supplied in the kit to yield a colorimetric product; absorbance is measured spectrophotometrically at the wavelength indicated by the kit protocol. Catalase activity was calculated from an H_2_O_2_ standard curve and is reported as U per mg protein (1 U = defined by the manufacturer’s unit definition). Samples were prepared and assayed in duplicate and blank-corrected.

Glutathione peroxidase activity was determined using the classical coupled assay of Paglia and Valentine. In this assay, GPx catalyzes the reduction in H_2_O_2_ (or an organic hydroperoxide) by reduced glutathione (GSH), forming GSSG; glutathione reductase then reduces GSSG back to GSH at the expense of NADPH. The oxidation of NADPH to NADP^+^ is monitored as a decrease in absorbance at 340 nm. Practically, assays were run in a cuvette or microplate containing the phosphate buffer, GSH (substrate), excess glutathione reductase, NADPH, and a defined concentration of H_2_O_2_ to initiate the reaction. The linear rate of decline in A_340_ (ΔA_340_/min) was converted to µmol NADPH oxidized per minute using the molar extinction coefficient for NADPH (6.22 × 10^3^ M^−1^·cm^−1^, adjusted for path length), and activity was expressed as U per mg protein (1 U = 1 µmol NADPH oxidized min^−1^ under assay conditions). All GPx assays were performed at controlled temperatures, with appropriate blanks (no substrate/no enzyme) and run in duplicate. Protein normalization used the same BCA measurements described above [48].

### 2.12. Immunohistochemical Evaluation of GFAP and IBA-1

Paraffin-embedded tissue sections were deparaffinized, rehydrated, and incubated in 0.3% hydrogen peroxide in methanol to block endogenous peroxidase activity. Samples were then permeabilized with 0.1% Triton X-100 and subsequently blocked with 2% normal goat serum, followed by biotin/avidin blocking using reagents from Vector Laboratories (Newark, CA, USA). The sections were incubated overnight at 4 °C with primary antibodies against GFAP or IBA (1:100; Santa Cruz Biotechnology, Dallas, TX, USA). After rinsing in PBS, the slides were treated with HRP-conjugated secondary antibodies specific to the host species (1:2000; Jackson ImmunoResearch, West Grove, PA, USA) and developed using the avidin–biotin–peroxidase complex (Vector Laboratories). Images were captured with a Leica DM6 microscope and analyzed using the ImageJ software. DAB staining intensity was quantified through color deconvolution and the IHC Profiler plug-ins, and results were reported as pixel intensity values. All evaluations were conducted independently by two investigators blinded to the experimental groups.

### 2.13. Immunofluorescence of ZO-1 and Occludin

Colon and brain tissue sections were incubated overnight at 37 °C in a humidified chamber with primary antibodies targeting ZO-1 or occludin (1:100; Santa Cruz Biotechnology). Following PBS washing, samples were incubated for 1 h at 37 °C with the corresponding secondary antibodies: Alexa Fluor-594-labeled anti-mouse IgG (1:1000; Molecular Probes, Wokingham, UK) or Alexa Fluor-488-labeled anti-mouse IgG (1:2000; Molecular Probes, UK). Nuclear staining was performed using DAPI (2 µg/mL in PBS; Hoechst, Frankfurt, Germany). Images were captured using a Leica DM6 fluorescence microscope, digitized, and processed according to previously established analytical protocols.

### 2.14. ELISA Determination for Serum Citokynes and Corticosterone

Cytokine concentrations (TNF-α, IL-1β, IL-6, and IL-10) were quantified using commercially available ELISA kits, following the protocols provided by the manufacturers, as described previously [49,50]. Serum corticosterone concentrations were determined using a commercial ELISA kit (Arbor Assays, Ann Arbor, MI, USA; catalog no. K014) according to the manufacturer’s guidelines. Samples were diluted 1:150 and assayed in duplicate. Following dilution, a dissociation buffer was added and the samples were incubated at 60 °C. Absorbance was recorded at 450 nm with a microplate reader (Victor V3, PerkinElmer, Waltham, MA, USA). Corticosterone levels were calculated from a standard curve [51,52].

### 2.15. Statistical Evaluation

All assessments and data analyses were conducted by investigators blinded to the experimental group allocations to minimize bias. Results are expressed as mean ± SEM and are based on a minimum of three independent experiments performed on separate days. Sample sizes for in vivo studies were determined using the G*Power 3.1 software (Heinrich-Heine-University, Düsseldorf, Germany). No subjects were excluded from the analysis. Statistical evaluation was carried out using two-way ANOVA followed by Bonferroni’s post hoc test for multiple comparisons. A *p*-value ≤ 0.05 was considered statistically significant.

## 3. Results

### 3.1. Vitis vinifera Supplementation Ameliorates TBI-Induced Deficits in Spatial Learning, Memory, Anxiety-like Behavior, Locomotor Activity, and Corticosterone Levels

**Figure 1:**
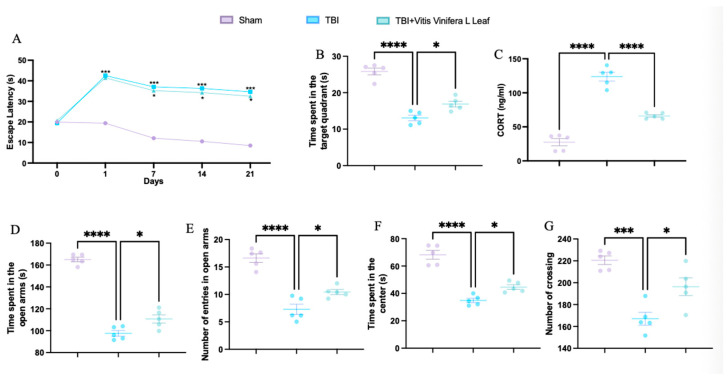
Effects of *Vitis vinifera* on spatial learning, memory function, anxiety, and locomotor activity. Morris water maze training and probe (**A**,**B**); corticosterone serum levels (**C**); elevated plus maze test (**D**,**E**); open field (**F**,**G**). A *p*-value of < 0.05 is considered significant; the data are presented as mean ± SEM. * *p* < 0.05 vs. TBI; *** *p* < 0.001 vs. Sham; **** *p* < 0.001 vs. TBI.

TBI significantly increased escape latency in the Morris water maze compared with the sham group, while treatment with *Vitis vinifera* L. leaf extract reduced latency, indicating improved spatial learning (Figure 1A). In the probe trial, TBI animals spent less time in the target quadrant, whereas treatment increased the time spent, suggesting enhanced memory performance (Figure 1B). Serum corticosterone levels were elevated in the TBI group and significantly reduced following *Vitis vinifera* L. leaf extract administration (Figure 1C). In the elevated plus maze, TBI decreased both the time spent and the number of entries in the open arms—effects that were partially reversed by treatment (Figure 1D,E). In the open field test, TBI animals exhibited reduced time spent in the center and fewer crossings, while *Vitis vinifera* L. leaf extract increased both parameters, indicating reduced anxiety-like behavior and improved locomotor activity (Figure 1F,G).

### 3.2. Daily Vitis vinifera Supplementation Reduces Cerebral Edema and Infarct Volume and Promotes Histological Recovery Following TBI

**Figure 2:**
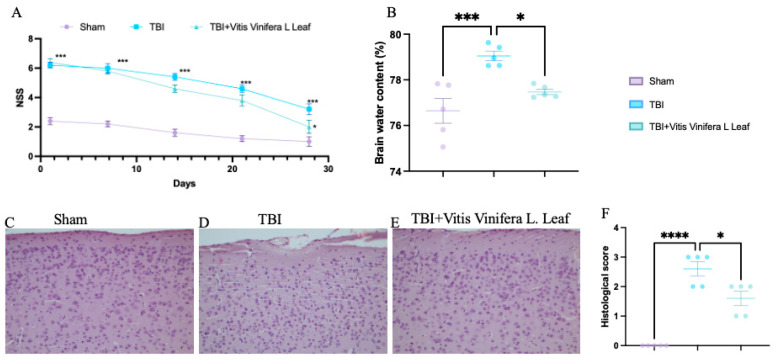
Effect of *Vitis vinifera* on TBI-induced brain injury. Brain water content in the experimental groups (**A**); neurological severity score at indicated time post-TBI (**B**); H&E brain staining: sham (**C**), TBI (**D**), *Vitis vinifera* (**E**) group; histological score (**F**). A *p*-value of < 0.05 is considered significant; the data are presented as mean ± SEM. * *p* < 0.05 vs. TBI; *** *p* < 0.001 vs. Sham; **** *p* < 0.001 vs. TBI.

**Figure 3:**
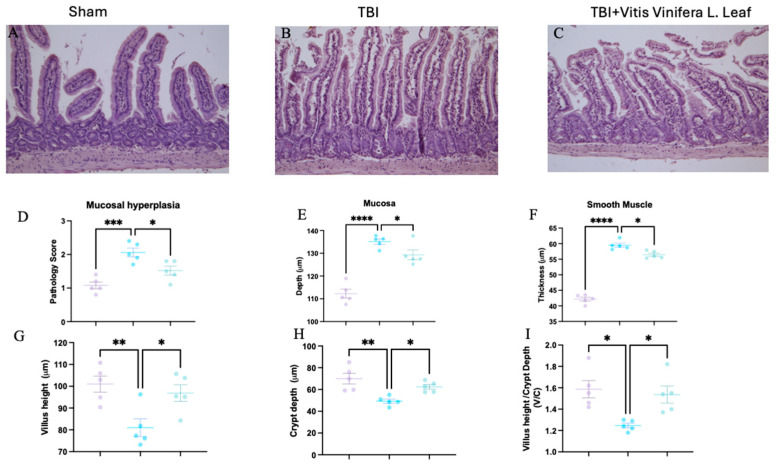
Effect of *Vitis vinifera* on TBI-induced intestine injury. H&E staining: sham (**A**), TBI (**B**), *Vitis vinifera* (**C**) group; mucosal hyperplasia (**D**); mucosal depth (**E**); smooth muscle (**F**); Villus height (**G**); Crypt depth (**H**); and ratio Villus/crypt (**I**). A *p*-value of < 0.05 is considered significant; the data are presented as mean ± SEM. * *p* < 0.05 vs. TBI; ** *p* < 0.01 vs. TBI; *** *p* < 0.001 vs. TBI; **** *p* < 0.0001 vs. TBI.

TBI significantly increased neurological severity scores (NSSs) compared with the sham group, indicating pronounced neurological impairment (Figure 2A). Treatment with *Vitis vinifera* L. leaf extract markedly reduced NSS values over time (*p* < 0.05), suggesting improved functional recovery. Brain water content was elevated in the TBI group compared with sham controls (** *p* < 0.001), whereas the *Vitis vinifera* L. leaf extract significantly reduced edema (*p* < 0.05; Figure 2B). Histological analysis revealed neuronal degeneration and tissue disorganization in the TBI group (Figure 2D), while the *Vitis vinifera* L. leaf extract treatment preserved cortical architecture and reduced cellular damage (Figure 2E). Quantitative evaluation confirmed a significant increase in histological score after TBI (*** *p* < 0.0001), which was ameliorated by the *Vitis vinifera* L. leaf extract (*p* < 0.05; Figure 3F).

### 3.3. Vitis vinifera Limits Intestinal Histological Alteration Induced by TBI

Histological analysis of intestinal sections showed marked structural alterations following TBI. Compared with the sham group (Figure 3A), the TBI group exhibited mucosal hyperplasia, villus shortening, and crypt deepening (Figure 3B). Treatment with the *Vitis vinifera* L. leaf extract partially restored normal architecture, reducing mucosal damage (Figure 3C). Quantitative analysis revealed that TBI significantly increased the pathology score (Figure 3D), mucosal depth (Figure 3E), and smooth muscle thickness (Figure 3F) compared with the sham group (** *p* < 0.001 to *** *p* < 0.0001). The *Vitis vinifera* L. leaf extract significantly reduced these alterations (*p* < 0.05). Similarly, villus height was decreased and crypt depth increased after TBI (Figure 3G,H), resulting in a lower villus height/crypt depth ratio (Figure 3I). Treatment with the *Vitis vinifera* L. leaf extract significantly improved these parameters (*p* < 0.05), indicating attenuation of intestinal structural injury.

### 3.4. Vitis vinifera Reduces Astrogliosis and Microgliosis

**Figure 4:**
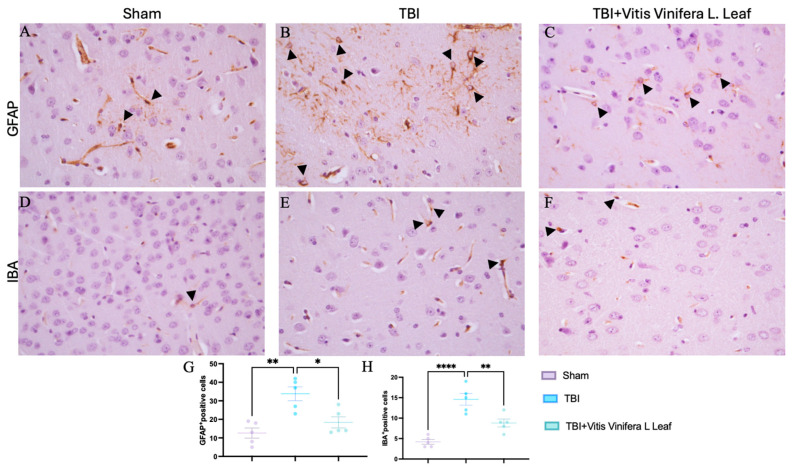
Effects of *Vitis vinifera* supplementation of neuroinflammation. Immunoistochemistry of GFAP in Sham (**A**), TBI (**B**), and *Vitis vinifera* (**C**). Immunoistochemistry of Iba in Sham (**D**), TBI (**E**), and *Vitis vinifera* (**F**). Densitometric analysis of positive pixel for GFAP (**G**) and IBA (**H**). A *p*-value of < 0.05 is considered significant; the data are presented as mean ± SEM. * *p* < 0.05 vs. TBI; ** *p* < 0.01 vs. TBI; **** *p* < 0.0001 vs. TBI. Black arrows indicates positive cells.

Immunohistochemical analysis was performed to evaluate glial activation in the cerebral cortex using glial fibrillary acidic protein (GFAP) as a marker of astrocytes and ionized calcium-binding adapter molecule 1 (IBA-1) as a marker of microglia (Figure 4A–F). In the Sham group, GFAP-positive astrocytes exhibited thin processes and sparse distribution (Figure 4A). In contrast, the TBI group showed a marked increase in GFAP immunoreactivity with hypertrophic astrocytes and dense staining patterns (Figure 4B), indicating pronounced astrogliosis. Dayli administration with the *Vitis vinifera* L. leaf extract notably reduced GFAP expression compared with the TBI group (Figure 4C, see densitometric analysis Figure 4G). Similarly, IBA-1 immunostaining showed resting microglia with small cell bodies and ramified morphology in the Sham group (Figure 4D). Following TBI, IBA-1-positive microglia were markedly increased and displayed activated morphology with enlarged somata and thickened processes (Figure 4E). Dayli supplementation of the *Vitis vinifera* L. leaf extract reduced the density and activation of IBA-1-positive cells compared with the TBI group (Figure 4F, see densitometric analysis Figure 4H).

### 3.5. Daily Administration of Vitis vinifera Improves Physiological Antioxidant Defense

**Figure 5:**
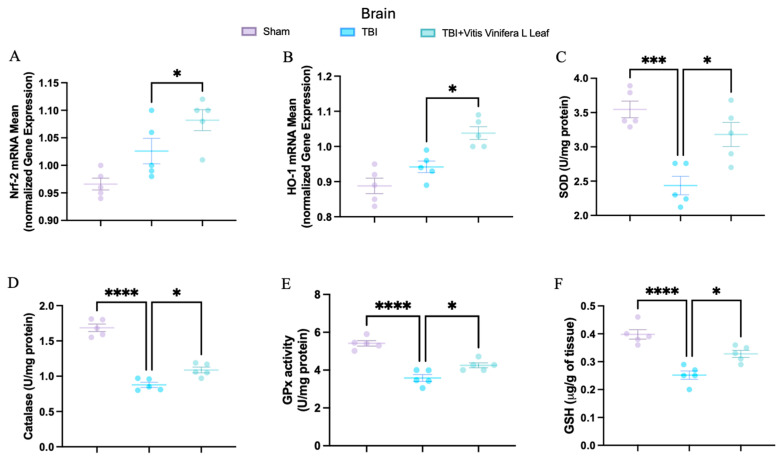
Effects of *Vitis vinifera*, on the mRNA levels in brain of Nrf-2 (**A**), HO-1 (**B**), SOD (**C**), Catalase (**D**), GPx activity (**E**), and GSH (**F**). A *p*-value of < 0.05 is considered significant; the data are presented as mean ± SEM. * *p* < 0.05 vs. TBI; *** *p* < 0.001 vs. TBI; **** *p* < 0.001 vs. TBI.

**Figure 6:**
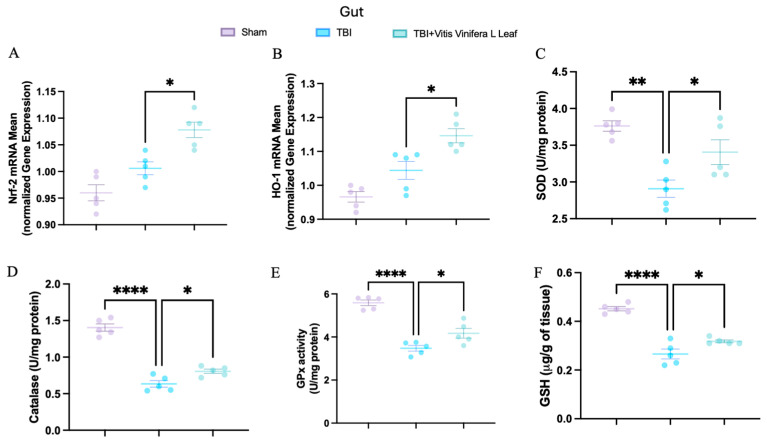
Effects of *Vitis vinifera*, on the mRNA levels in gut of Nrf-2 (**A**), HO-1 (**B**), SOD (**C**), Catalase (**D**), GPx activity (**E**), and GSH (**F**). A *p*-value of < 0.05 is considered significant; the data are presented as mean ± SEM. * *p* < 0.05 vs. TBI; ** *p* < 0.01 vs. TBI; **** *p* < 0.001 vs. TBI.

To evaluate oxidative stress, key mediators of antioxidant defense were assessed in both brain and intestinal tissues. Changes in the expression of antioxidant-related genes following VVL supplementation are presented in Figure 5 and Figure 6. No significant differences in gene expression were detected between the TBI and sham groups. However, daily VVL supplementation resulted in increased mRNA expression of Nrf2 (Figure 5A for brain and Figure 6A for gut), which is a master transcriptional regulator of antioxidant responses, and HO-1 (Figure 5B for brain and Figure 6B for gut), which is a downstream antioxidant enzyme, relative to sham controls.

In parallel, the activity of major antioxidant enzymes—including superoxide dismutase (SOD) (Figure 5C for brain and Figure 6C for gut), catalase (CAT) (Figure 5D for brain and Figure 6D for gut), and glutathione peroxidase (GPx) (Figure 5E for brain and Figure 6E for gut)—as well as glutathione (GSH) (Figure 5F for brain and Figure 6F for gut) levels, was quantified. TBI markedly reduced the activity of SOD, CAT, and GPx, along with GSH content, in both brain and intestinal tissues, consistent with increased oxidative burden and disrupted redox homeostasis. VVL supplementation effectively reversed these deficits, restoring antioxidant enzyme activities and GSH concentrations to values comparable to or, in some cases, indistinguishable from those observed in sham animals.

### 3.6. Vitis vinifera Supplementation Strengthens the Integrity of the Barrier After Chronic TBI

**Figure 7:**
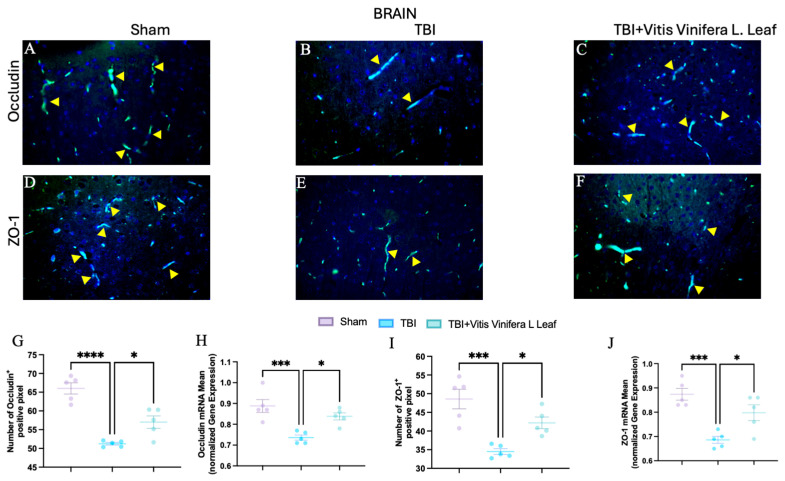
Effects of *Vitis vinifera* daily supplementation on ZO-1 and Occludin in brain. Immunofluorescence of Occludin in Sham (**A**), TBI (**B**), and *Vitis vinifera* (**C**). Immunofluorescence of ZO-1 in Sham (**D**), TBI (**E**), and *Vitis vinifera* (**F**). Densitometric analysis of Occludin (**G**) and mRNA (**H**). Densitometric analysis of ZO-1 (**I**), and mRNA (**J**). A *p*-value of < 0.05 is considered significant; the data are presented as mean ± SEM. * *p* < 0.05 vs. TBI; *** *p* < 0.001 vs. Sham; **** *p* < 0.001 vs. TBI. Yellow arrows indicates positive cells.

**Figure 8:**
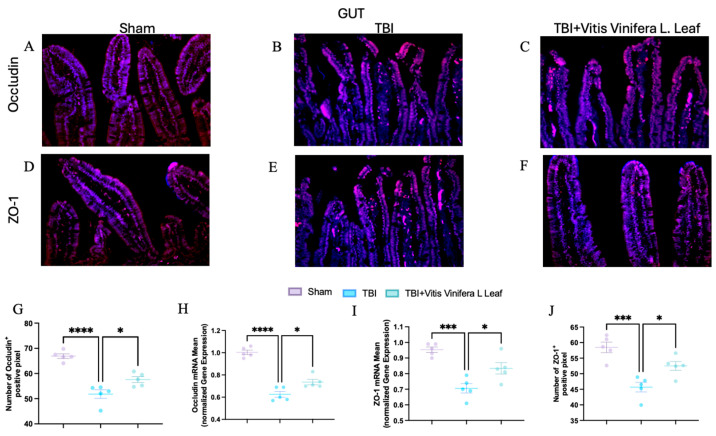
Effects of *Vitis vinifera* daily supplementation on ZO-1 and Occludin in gut. Immunofluorescence of Occludin in Sham (**A**), TBI (**B**), and *Vitis vinifera* (**C**). Immunofluorescence of ZO-1 in Sham (**D**), TBI (**E**), and *Vitis vinifera* (**F**). Densitometric analysis of Occludin (**G**) and mRNA (**H**). Densitometric analysis of ZO-1 (**I**) and mRNA (**J**). A *p*-value of < 0.05 is considered significant; the data are presented as mean ± SEM. * *p* < 0.05 vs. TBI; *** *p* < 0.001 vs. Sham; **** *p* < 0.001 vs. TBI.

To assess the effect of *Vitis vinifera* L. leaf extract on blood–brain barrier integrity, occludin and ZO-1 expression were evaluated by immunofluorescence and qPCR (Figure 7A–J and Figure 8A–J) in both the brain (Figure 7) and gut (Figure 8). In the Sham group, occludin and ZO-1 showed strong, continuous staining (Figure 7A,D for brain and Figure 8A,D for gut). TBI markedly reduced the fluorescence intensity and continuity of both proteins (Figure 7B,E for brain and Figure 8B,E for gut), indicating tight junction disruption. Daily administration with *Vitis vinifera* L. leaf extract partially restored occludin and ZO-1 expression (Figure 7C,F for brain and Figure 8C,F for gut). Quantitative analysis confirmed significant decreases in occludin and ZO-1-positive pixels and mRNA levels in the TBI group compared with Sham (** *p* < 0.0001, * *p* < 0.001). Administration of *Vitis vinifera* L. leaf extract significantly increased both protein and gene expression compared with TBI (*p* < 0.05) (Figure 7G–J for brain and Figure 8G–J for gut).

### 3.7. Vitis vinifera L. Leaf Extract Attenuates Systemic Inflammation

**Figure 9:**
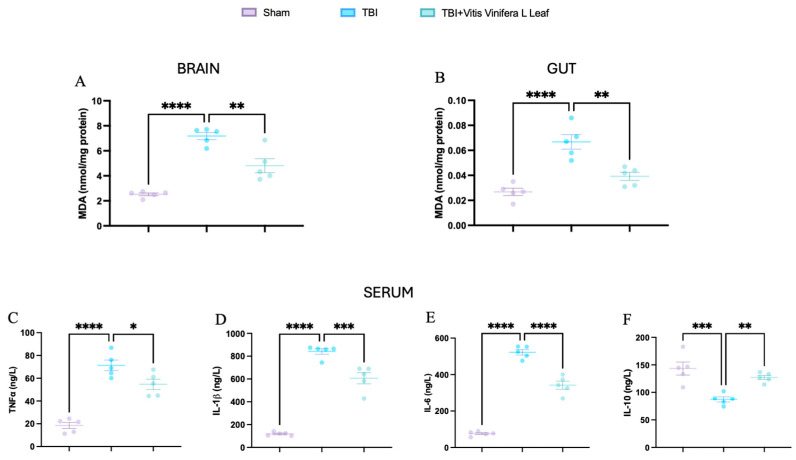
Effects of *Vitis vinifera* daily supplementation systemic inflammation. MDA on brain (**A**) and gut (**B**). Serum cytokines levels of TNF-α (**C**), IL-1β (**D**), IL-6 (**E**), and IL-10 (**F**). A *p*-value of < 0.05 is considered significant; the data are presented as mean ± SEM. * *p* < 0.05 vs. TBI; ** *p* < 0.01 vs. TBI; *** *p* < 0.001 vs. Sham; **** *p* < 0.001 vs. TBI.

Malondialdehyde (MDA) levels were measured to assess oxidative stress in the brain and gut (Figure 9A,B). TBI significantly increased MDA concentrations compared with the Sham group (** *p* < 0.0001), indicating elevated lipid peroxidation. Treatment with *Vitis vinifera* L. leaf extract markedly reduced MDA levels in both tissues (*p* < 0.01), suggesting attenuation of oxidative damage. Serum cytokine analysis further revealed a pronounced systemic inflammatory response following TBI (Figure 9C–F). Levels of TNF-α, IL-1β, and IL-6 were significantly elevated compared with Sham (** *p* < 0.0001), whereas IL-10, which is an anti-inflammatory cytokine, was significantly decreased (* *p* < 0.001). Administration of *Vitis vinifera* L. leaf extract significantly lowered the pro-inflammatory cytokines and restored IL-10 levels (*p* < 0.05–0.01).

## 4. Discussion

Daily oral supplementation with bioactive natural compounds represents a promising strategy to counteract the multifaceted damage induced by traumatic brain injury (TBI). TBI sets in motion an acute mechanical insult—manifested as cortical contusion, axonal shearing, and intracerebral hemorrhage—followed by a secondary cascade of excitotoxicity, mitochondrial dysfunction, calcium dysregulation, and excessive reactive oxygen species (ROS) production. These secondary processes sustain oxidative stress and neuroinflammation, disrupt the blood–brain barrier (BBB), and extend injury beyond the brain through systemic pathways. One key route of systemic propagation is the gut–brain axis, which is a bidirectional network integrating neural, immune, endocrine, and microbial signals. Post-TBI, increased intestinal permeability and loss of epithelial tight junction integrity facilitate the translocation of pro-inflammatory mediators into circulation, amplifying neuroinflammation and contributing to behavioral and cognitive deficits. Oxidative stress acts as a unifying driver of both central and peripheral injury, making antioxidant-targeted interventions an attractive therapeutic avenue. In this study, we investigated the effects of daily supplementation with VVL extract—a polyphenol-rich natural formulation containing resveratrol, catechins, and anthocyanins. These compounds are known to activate the Nrf2/HO-1 antioxidant pathway, suppress ROS, and stabilize tight junction proteins such as ZO-1 and occludin, thereby protecting both neurovascular and intestinal barriers. Our findings reveal that daily VVL supplementation significantly improves behavioral outcomes after TBI. In the Morris water maze, VVL-treated animals exhibited shorter escape latencies and greater target quadrant occupancy, indicating enhanced spatial learning and memory. In the elevated plus maze and open field tests, supplementation reduced anxiety-like behaviors and restored exploratory activity. Histological analyses showed that daily VVL intake mitigated neuronal degeneration, edema, and cortical disorganization, while preserving intestinal villus morphology and reducing inflammatory infiltration. Biochemically, supplementation reversed TBI-induced suppression of superoxide dismutase (SOD), catalase (CAT), glutathione peroxidase (GPx), and glutathione (GSH) in both brain and gut tissues. This recovery was accompanied by robust upregulation of Nrf2 and HO-1 mRNA, which is consistent with the activation of endogenous antioxidant defenses. Furthermore, daily VVL administration restored ZO-1 and occludin expression in both CNS and intestinal tissues, indicating repair of BBB and gut barrier integrity—an effect likely contributing to the attenuation of systemic inflammatory signaling via the gut–brain axis. Additionally, daily supplementation with *Vitis vinifera* L. shows an important reduction in the systemic inflammation in TBI induced by the reduction in serum cytokines. Overall, this work positions daily oral supplementation with a polyphenol-rich natural extract as a holistic, multi-target therapeutic approach for TBI. By activating antioxidant pathways, reinforcing barrier integrity, and modulating neuro-inflammatory cross-talk between the gut and brain, VVL demonstrates the potential to improve cognitive, structural, and biochemical outcomes. These findings provide a strong rationale for integrating targeted nutritional supplementation into post-TBI management and warrant further investigation into its specific bioactive constituents, pharmacokinetics, and long-term benefits.

## 5. Conclusions

While this study demonstrates that daily supplementation with VVL can mitigate multiple central and peripheral consequences of TBI, certain limitations should be considered. First, the work was performed in a preclinical rodent model, which, although recapitulating many aspects of human TBI, cannot fully capture the complexity, heterogeneity, and comorbidities present in patients. Second, supplementation was initiated immediately after injury and administered daily under controlled conditions; real-world scenarios may involve delayed intervention, variable adherence, and differences in baseline nutritional status. Third, the observation period was limited to the acute and subacute phases, leaving the long-term effects of chronic VVL supplementation on cognitive function, structural preservation, and systemic physiology unknown. Future studies should expand on these findings by assessing the efficacy of daily VVL supplementation in delayed treatment paradigms and over extended recovery periods, including chronic stages of TBI. Clinical investigations will be essential to evaluate safety, tolerability, and optimal dosing strategies in diverse patient populations. In addition, exploring the integration of VVL into multimodal therapeutic regimens—combining nutritional supplementation with rehabilitation, pharmacological agents, and microbiome-targeted strategies—could enhance recovery outcomes. Given its broad impact on antioxidant defense, barrier integrity, and behavioral performance, daily VVL supplementation holds promise not only for TBI management but also for other disorders involving oxidative stress, systemic inflammation, and gut–brain axis dysfunction.

## Data Availability

The original contributions presented in this study are included in the article. Further inquiries can be directed to the corresponding author.
